# Implications of electricity and gas price coupling in US New England region

**DOI:** 10.1016/j.isci.2023.108726

**Published:** 2023-12-13

**Authors:** Qiwei Zhang, Fangxing Li, Xin Fang, Jin Zhao

**Affiliations:** 1 Department of Electrical Engineering and Computer Science, The University of Tennessee, Knoxville, TN 37996, USA; 2 Department of Electrical and Computer Engineering, Mississippi State University, Mississippi State, MS 39762, USA; 3 Department of Electronic and Electrical Engineering, School of Engineering, Trinity College Dublin, D02PN40 Dublin, Ireland

**Keywords:** Electricity, Electrical engineering, Energy Modelling

## Abstract

The tight coupling between electricity and gas has put the US New England region at constant risk of electricity price spikes due to a shortage of gas supplies, especially in the wake of limited natural gas supply in 2022. Here, we investigate the electricity-gas price couplings in the six states in New England from 2006 to 2022. We found that the price coupling in New England has been high and consistent in the past five years across all states, despite varying levels of gas-fired power generation. Additionally, we anticipate it will remain high even with increasing renewables by 2030. Furthermore, the price coupling exhibits an asymmetrical influence with electricity prices closely tracking gas prices, while gas prices are weakly affected by electricity price variations. Our findings also suggest that promoting electricity-gas cooperations could potentially mitigate the asymmetrical influence and electricity price spikes in New England.

## Highlights

• High electricity-gas price coupling is observed across all states in New England
• Increasing renewable generations has not decreased the electricity-gas price coupling in New England
• The coupling between electricity and gas prices is asymmetrical
• The advancement of electricity-gas cooperation potentially improves the asymmetrical price coupling

## Graphical abstract

**Figure:**
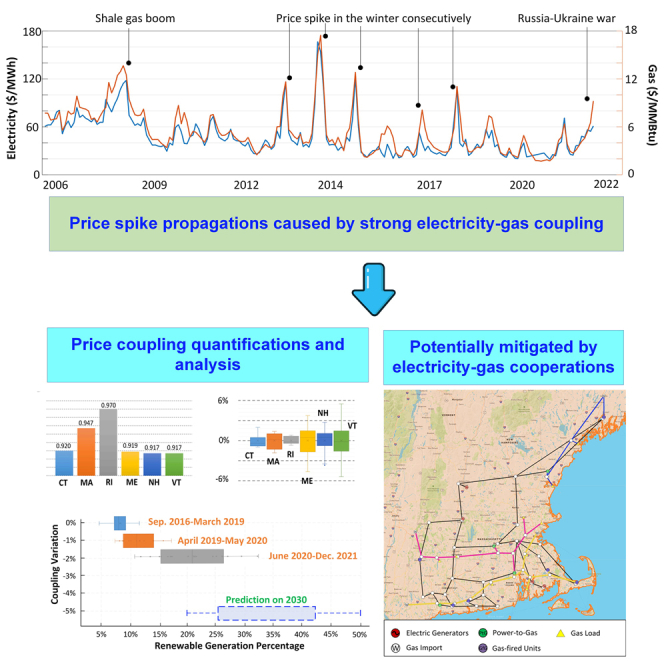

## Introduction

The continued COVID-19 pandemic and the recent Russian-Ukraine war have been dragging down the global economy and curtailing natural gas production.^1^ Gas prices have become extremely volatile, with prices experiencing the biggest price swing in the past two decades.^2^ The New England region of the US consistently depends on importing liquefied natural gas from high-priced tight global spot market to support its electricity supply, with gas units playing a continual role in determining electricity prices. The surge in natural gas prices has led to a significant increase in electricity costs within the New England region. Specifically, the wholesale electricity price in New England soared to $137/MWh in the first three months of 2022, showing an 83% increase compared with $75/MWh in the same period of 2021.^3^ Such propagation of price spikes underscored a strong coupling between electricity and gas prices in New England and has started some debates about the promotion of gas unit.^4^^,^^5^ The increase of electricity bills is an annual trend in New England due to surging natural gas prices, as observed in both 2020 and 2021. This trend become especially evident in 2022 that electricity priced are skyrocketing with tight gas supplies. This gives a stark reminder of New England's reliance on natural gas units highlighting the need to restrict the price spike propagated from gas to electricity by reducing the strong coupling between electricity and gas. To provide a quantitative basis for this noteworthy linkage between electricity and gas prices in New England, this paper delves into electricity and gas prices across the six states within the New England region. Various previous studies have examined the benefit of natural gas units in the electricity sector. The natural gas-fired generation has been considered one of the most economical ways to reduce emissions and move toward a decarbonized power grid,^6^^,^^7^^,^^8^^,^^9^ and it has been considered an efficient way to provide operational flexibility to support renewable integrations.^10^^,^^11^^,^^12^^,^^13^ However, other studies raised concerns about the strong coupling between electricity and gas systems, such as energy security,^14^^,^^15^^,^^16^^,^^17^ financial risk,^18^^,^^19^ and delay of renewable energy transitions.^20^^,^^21^ New England, where electricity generation and gas consumption are strongly intertwined, has been continuously receiving benefits and suffering issues from the tight coupling between electricity and gas.^22^^,^^23^^,^^24^^,^^25^^,^^26^^,^^27^ In the light of these discussions, we examined electricity and gas prices in the six New England states: Connecticut (CT), Maine (ME), Massachusetts (MA), New Hampshire (NH), Rhode Island (RI), and Vermont (VT), from 2006 to 2021, and we have the following three new observations.

1) Strong electricity-gas price coupling is evident in both areas with high gas-fired generation (e.g., in MA) and areas with low gas-fired generation (e.g., in VT) due to the interconnected electric grid.
2) Renewable generations are gradually reshaping the generation mix in New England, but we have only observed a small decrease (less than 3%) in electricity-gas price coupling over the last five years. We also observed a staircase relationship between the variation of coupling and the increasing renewable penetration. The electricity-gas coupling gradually moves down the staircase with higher renewable generations, although the decreased coupling is small.
3) Further, our analysis shows an asymmetrical influence between electricity and gas prices, although the share of gas consumption from the electric sector is as high as the share of electricity generation from gas units in New England. The electricity price variations are dominated by gas price variations, instead of the operational condition of the electric grid, while electricity price variations have a small impact on gas prices.

## Results

### Coupling between electricity and gas prices in six states of New England

**Figure 1:**
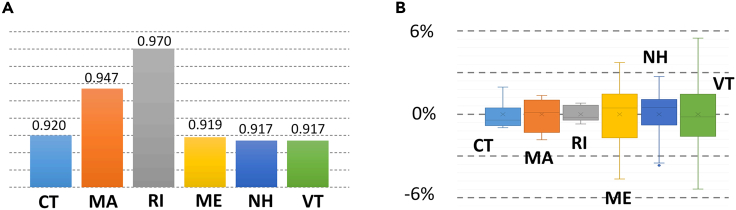
Electricity-gas price coupling in New England (A) Pearson correlation between the value of electricity and gas prices in the six states of New England. (B) Variation of the Pearson correlation value.

The electricity-gas price coupling is measured by Pearson correlation values, as shown in Figure 1A The coupling values for CT, MA, and RI are very high showing highly correlated electricity and gas prices. This strong correlation aligns with the high percentage of gas-fired electricity in overall electricity generation (i.e., 43.4%, 63.3%, and 95.4%) and the high percentage of gas consumed in electric sector in overall gas consumptions (i.e., 50.2%, 41.6%, and 60.3%) in the three states. However, despite having much lower percentage of gas-fired electricity generation (33.2% in NH and 23.6% in ME), the price coupling in NH and ME is comparable to that in CT, MA, and RI. Therefore, even with a lower reliance on gas-fired units, NH and ME still experience a strong electricity-gas price coupling. This trend is further highlighted in VT, where the gas generation and gas consumption by the electric sector are almost negligible (i.e., both less than 0.1%), yet the electricity-gas price coupling remain significant (i.e., 0.917). When gas-fired unit accounts for a high share of electricity generation, the correlation value goes higher. The correlation value grows from 0.92 in CT (i.e., 43.4%) to 0.94 in MA (i.e., 63.3%), and to 0.97 in RI (i.e., 95.4%). When gas-fired electricity accounts for a low share of electricity generation, the correlation value will still be high and similar. The correlation values at CT, ME, NH, and VT are all around 0.919.

The evident electricity-gas price coupling in NH, ME, and VT indicates that strong electricity-gas price coupling exists, regardless of the share of gas-fired electricity generation. The interconnected electric grid propagates electricity-gas price coupling, as well as gas price spikes, from areas with a high share of gas-fired generation to areas with a low share of gas-fired generation. The electricity price is set by the scheme of locational marginal price. When the transmissions are operated with no congestion, the price at the electric consumption site is typically not influenced by the price of local generators. Instead, the electricity price is determined by the costliest dispatched unit in the system. This implies that the electricity price could be high even when nearby local units are cheap. The price pike that happened in MA, CT, and RI at the end of 2021, inevitably caused price spikes in VT, although gas-fired generation and gas consumption are very low in VT. VT has been relying on electricity imports since the shutdown of nuclear plants in 2014 (Table S2). However, we observed that the electricity-gas price coupling in VT was similar before and after 2014 (Table S2). In other words, the electricity-gas price coupling in VT has not been affected by the change in the resource mix. This result also implies that the marginal units in New England are generally located in states where gas generation is high (i.e., MA, CT, and RI), although the location of marginal units is not disclosed to the public. The variation of the electricity-gas price coupling value for the last five years was obtained for all six New England states, as shown in Figure 1B. Although electricity and gas prices have changed constantly over the past five years, most of the variations are less than 3% showing that the electricity-gas price coupling is high and consistent across the entire New England region.

### Impact of increasing renewable penetration on electricity-gas price coupling

**Figure 2:**
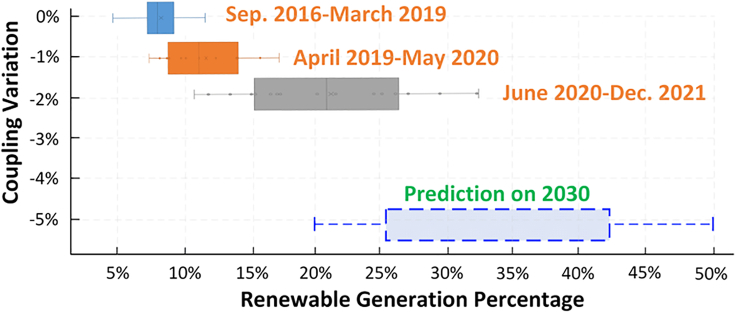
The variation of coupling under increasing renewable generations

New England has set aggressive targets for increasing renewable penetration to reduce 80% of greenhouse emissions by 2050.^28^ Our results report a staircase relationship between renewable penetration and price coupling. The price coupling will slowly step down over time as renewable generation increases, although the decreased amount has been low (see Figure 2). We analyzed and predicted the impact of renewable on price coupling based on the MA area (result for other areas can be found at Table S3). All renewable generation and price couplings from the last five years are grouped into 3 clusters. The length of the staircase gradually increases from cluster 1 to cluster 3, while the width of the staircase is similar. The staircase reflects the following two phenomena.

• First, the intermittent characteristics of renewable resources discord renewable generation from renewable penetration. From 2016 to 2021, the maximum renewable generation increased from 12% to 33%, but the minimal renewable generation only increased from 2% to 8%.
• Second, the decreased electricity-gas coupling is limited while renewable generation has significantly changed. The maximum renewable generation increased from 12% in 2017 to 33% in 2021, but there was only a slight decrease of 2% in price coupling in comparison to the price coupling in 2017.

The significant change in the length and negligible change in the width of the staircase indicates that the increase in renewable cannot decrease the electricity-gas price coupling immediately. By regressing renewable generations with the coupling variation (Note S1), we predict that the price coupling will only experience approximately a 5% drop when MA hits its 50% renewable portfolio in 2030,^29^ as shown in the blue bar. Consequently, the dropped coupling in New England as a whole will likely be less than 5% since the price coupling in other areas is milder compared to MA. The limitation of the prediction is indicated in Limitation 1.

### Mutual impact analysis for electricity and gas prices

**Figure 3:**
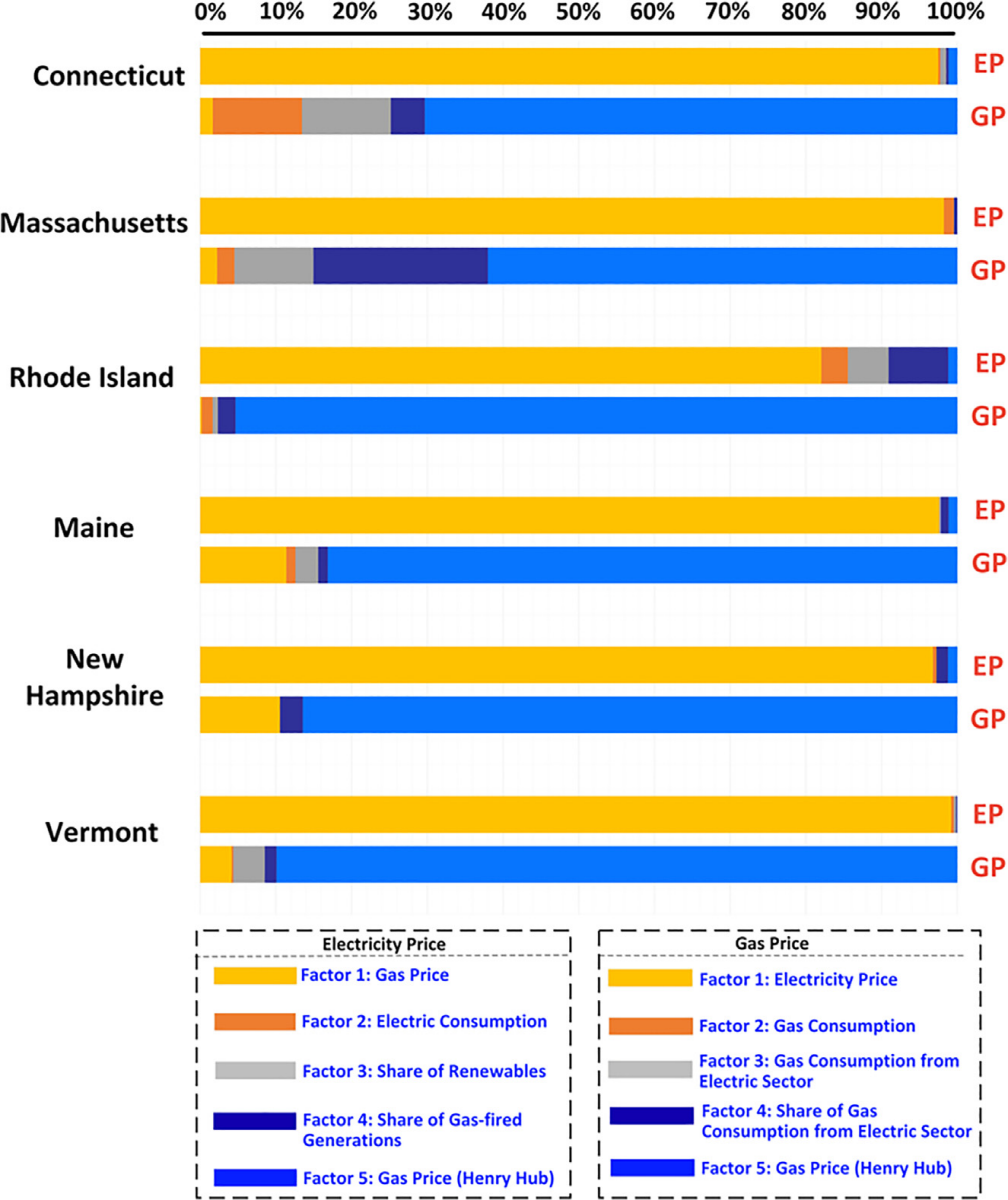
asymmetrical influence for electricity and gas price on operation data from March 2006 to December 2021

The observed strong electricity-gas price coupling could be attributed to the high reliance of electricity prices on gas prices, or vice versa. The impact of electricity price on gas price is not equal to the impact of gas price on electricity price (Table S7). To quantify the mutual influence between electricity and gas prices from both sides, we select the following factors that potentially affect electricity and gas price: gas/electricity prices, gas consumption, electricity loads, gas consumed by the electric sector, the share of renewable generation to meet the total load, the share of gas-fired generation to meet the total load, and gas price at Henry Hub reflecting the reference gas import price. Figure 3 shows the impact of a factor on electricity and gas prices in terms of percentage (details on the impact value can be found in the Note S2, and the limitation can be found in Limitation 2). The impact of gas prices on electricity prices is over 85%, overwhelming all the other factors in all six states. It shows that the electricity price in New England is generally determined by gas prices, consistent with causation analysis in Table S7. The impact value in Figure 3 shows that the electricity price is low when the natural gas price is low, and the electricity price is high when the natural gas price is high, regardless of other factors. The electric load, renewable generation, and gas-fired unit generation constantly change during the year 2006–2021. However, none of these changes in the electric sector are comparable to the impact that gas prices made on electricity prices. It reflects the fact that electricity prices in New England generally mirror the gas price, and the operating condition of the electric grid has a low impact on the electricity price. By contrast, the impact of electricity prices on gas prices is low in all six states (i.e., less than 10%). As shown in the stack bar, gas prices in New England mainly align with the reference gas price at Henry Hub (over 65% for all areas), while other factors have more limited impacts. Gas-fired generation accounted for most electricity in New England (i.e., 43.4%, 63.3%, 95.4%, 26%, and 32% for CT, MA, RI, NE, and NH, respectively), but the electric sector also accounts for most of the gas consumption (i.e., 50.2%, 41.6%, 60.3%, 41%, and 59% for CT, MA, RI, NE, and NH, respectively). Under a function of market supply and demand, the electric sector, as the largest demand for gas consumption in New England, has less impact on the gas price. This can also be seen in the gas price being high even when the electricity has low gas consumption (Figure S3). This asymmetric influence indicates that gas prices can push a price spike in electricity prices, even during the period of low gas consumption by the electric sector.

### The impact of electricity-gas coordination on electricity-gas price coupling

**Figure 4:**
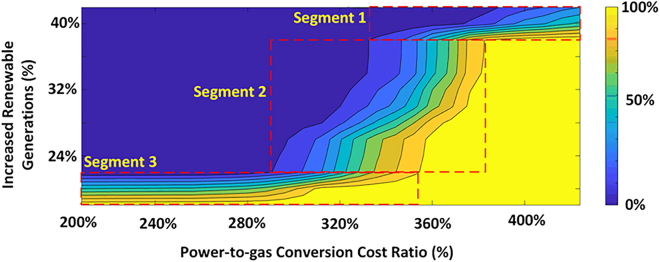
The variation of asymmetric influence between electricity and gas price under electricity-gas cooperation The x axis represents the PtG conversion cost describing the ratio between the PtG conversion cost and gas price at Henry Hub. The y axis represents the increased renewable generation indicating the added renewable generation to the existing renewable generation in terms of percentage. The orange and blue colors indicate the variation of asymmetric influence, as shown in the color bar on the left. The area with a red frame points out segments with different sensitivities to renewable energy and PtG cost.

**Figure 5:**
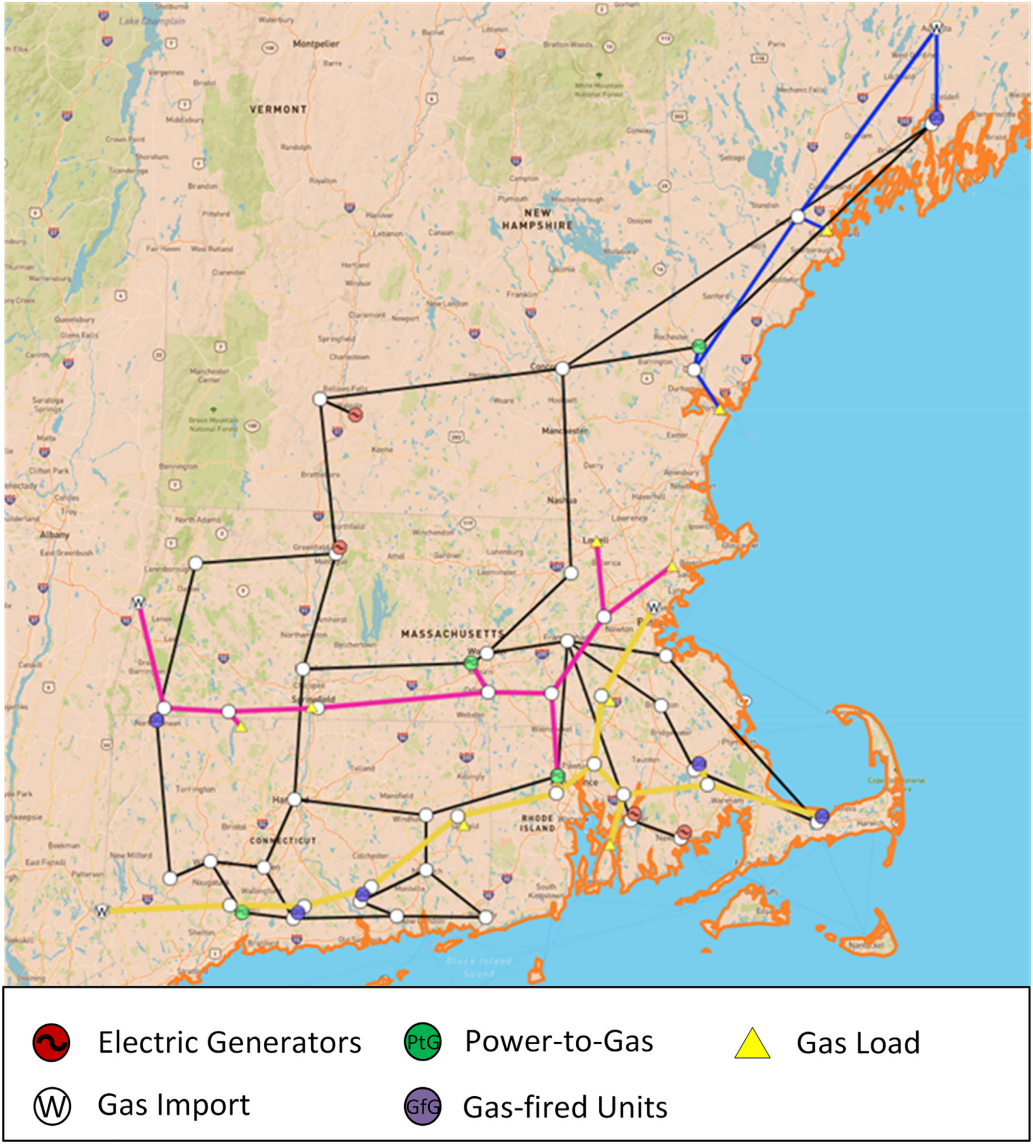
The simplified electric and gas test system for New England The topology and simplified systems are performed on the Large-scale Testbed (LTB)^31^^,^^32^ provided by the CURENT research center. Detailed description of the simulation system can be found in the supplementary file (Note S3).

Emerging power-to-gas (PtG) technologies are expected to enhance electricity and gas cooperation. Surplus electricity can be converted into hydrogen gas through electrolysis technology, which can be used directly or further converted to methane. The cooperation enables an efficient energy transition between electricity and natural gas systems through gas-fired units and PtG, which means the electricity sector will also be on the supply side of the gas sector. Figure 4 provides an analysis of the impact of electricity-gas coordination on the electricity-gas price coupling. The simulation is based on simplified New England electric and gas networks, as shown in Figure 5. Simulations are created based on the electric load, gas load, renewable generation, and gas import prices from 2006 to 2021 in New England. The limitation of the simulated results is discussed in Limitation 3 and Limitation 4. The simulation results show that electricity-gas cooperation has the potential to improve the current asymmetrical influence between electricity and gas prices, particularly with further renewable integration and PtG conversion cost reduction. The simulation study gives the following two observations.

• As shown in the blue area in Figure 4, when more than 30% of renewable generations are integrated and the PtG conversion cost drops below 320% of the gas import price, the asymmetric influence between electricity price and gas price becomes a symmetric influence. With sufficient renewable generation and low PtG conversion cost, the electricity price is able to adjust the gas price. This means that in the event of a gas price spike, the electricity price will adjust the local gas price, instead of having the gas price spike push up the electricity price. Although the PtG cost is significantly higher than today’s natural gas cost,^30^ this simulation results also suggest that, even if the PtG conversion cost remains relatively high in the future, electricity-gas coordination will mitigate price spike propagation with sufficient renewables. The effectiveness of electricity-gas coordination in mitigating the price spikes propagation will be contingent on the efficiency of the energy transition.
• The slopes of the contour lines show that the asymmetric influence varies with renewable generations and PtG cost under different sensitivities. In Segment 1, where the PtG cost ratio is very high, the asymmetric influence improves slowly even with high renewable energy. Similarly, in Segment 3, where renewable energy is insufficient, the asymmetric influence improves slowly even with low PtG cost. However, in Segment 2, the asymmetric influence is notably sensitive to both renewable energy and PtG cost. Therefore, aiming at using electricity-gas coordination to reduce the price spike propagation, there is an optimal development pace for renewable energy expansion and PtG cost reductions.

## Discussion and conclusion

Although areas with higher gas-fired electric generation have a higher coupling value in general, our results found that the states with low and almost no gas-fired generation still indicate an evident electricity-gas price coupling. This phenomenon indicates that when a gas price spike drives up the electricity price in areas with high gas-fired generation (e.g., MA, RI, and CT), it inevitably drives up the electricity price in nearby areas with low gas-fired generation (e.g., VT). The impact of the gas price spike is propagated by electric prices across New England. This observation implies an inequity in electricity pricing, wherein stakeholders like load aggregators in regions with low gas-fired generation bear the same risk and responsibility as these regions with large amounts of gas-fired generation. This suggests the need for policymakers to consider implementing new measures and pricing models designed to limit the asymmetrical influence of gas prices on electricity prices. For example, working papers^33^ propose to separate the market-clearing of green power, particularly renewables, from the conventional electricity market. This separation aims to mitigate the electricity-gas price coupling, allowing renewables to play a more significant role in determining electricity prices. Such an approach could be a potential solution to restrict the price spike propagation in New England.

Our analysis also shows a staircase relationship between the variation of electricity-gas price coupling and the increasing renewable penetrations, and the coupling will only experience a small drop with increasing renewable penetration in New England by 2030. This is primarily because the electricity price is set by the price of marginal units. Even with higher renewable generations, the marginal units will still be dominated by gas units, particularly in peak hours. The high electricity price brought by electricity-gas price coupling and marginal pricing scheme may be financially attractive for zero marginal cost renewables, but it is also worth noting that the unpredictable nature of renewable energy could bring renewable owners severe real-time risk under high electricity prices. However, the price volatility due to the strong electricity-gas price coupling could be financially attractive for energy storage systems. These phenomena call for more advanced risk-hedging strategies beyond the existing marginal price scheme. The expected small drop in the price coupling could be due to the low-demand hours when other units may have chances to set the price under higher renewable penetrations. In general, the variation of other fuels may have a very limited impact on the price coupling, especially given the fact that gas, nuclear, and renewable accounts for over 90% of electricity generation in New England. This may not be true for other states, such as North Dakota, where coal contributes to over 50% of electricity generation, implying that coal could function as marginal units sometimes.

Further, our analysis shows asymmetrical relationship between the gas price and the electric price in New England from 2006 to 2021. The electricity price is dominated by gas prices, including price spikes and low prices, while the electric sector has very little impact on gas prices. We found that the variation of gas consumption by the electric sector has a low ability to impact the gas price, although the share percentage of gas-fired generation in the electric sector is around the same level as the share of electricity-generating gas in the gas sector in New England. This asymmetrical influence between the electricity and gas prices suggests less stable energy financial networks in New England. The variations in gas prices directly impact electricity prices without any inherent mechanism to mitigate such price spike propagation. This means that no matter how the gas price spike discourages the use of gas-fired units, the electricity price consistently mirrors the gas price. Several previous analytic studies have affirmed or showed alignment with our findings in New England. For example, the discussion^34^ and studies^35^^,^^36^ support our findings that price spikes are transmitted between different electric regions and sufficient electric transmission capacity may cause a spillover effect of price spikes. Also, previous research works^27^^,^^37^^,^^38^^,^^39^ and reports^40^ argue that solely increasing renewable penetration has a limited impact on electricity-gas price coupling or a limited capability of mitigating the unilateral price spike propagation, which aligns with our second finding in New England.

Therefore, achieving a symmetrical influence is desirable from the perspective of mitigating the price spike propagation from gas to electricity. Under a symmetrical influence, the electricity price variation can also affect the gas price, rather than just following it, which potentially mitigates gas price spikes. As discussed in the research work,^41^ an asymmetrical system structure is more prone to financial contagions than a symmetrical system structure unless the financial shock exceeds a critical threshold. Several research works exploring systemic risk among gas and electricity systems also affirmed our findings, such as the spillover effect from gas to electricity being stronger than the spillover effect from electricity to gas,^42^ and an adverse event in the gas sector having a higher impact on the overall energy sector compared with an adverse event in the electric sector.^43^ The simulation study suggests that promoting electricity-gas cooperations could potentially establish a more symmetrical influence between gas prices and electricity prices. During a gas price spike, the local gas price could be determined by the cost of power units and the cost of PtG, provided that there is sufficient renewable generation and reasonable PtG costs.

The aforementioned observations and discussion give a reminder to New England policymakers that the existing electricity pricing scheme and technologies may prove insufficient under the fast-evolving renewables and constrained gas supply. Notably, as indicated in the report,^44^ New England is expected to remain approximately 20% gas-fired electricity by 2050 suggesting that the issue of price spike propagation may persist for the foreseeable future. In summary, the authors suggest three potential directions to mitigate the existing strong asymmetrical influence between electricity and gas prices in New England. (1) New technological developments, such as electricity-gas coordination, have the potential to establish a symmetrical influence. Through an efficient electricity-to-gas conversion, electricity price could become a variable to adjust the gas price, instead of purely variating with the gas prices. (2) New operational design, such as enhancement of the marginal pricing scheme, could potentially reduce the impact of gas price spikes on electricity prices. The existing marginal pricing scheme prioritizes the gas units in determining system-wide electricity prices, thereby propagating the impact of gas price spikes. New designs may distribute the responsibility equitably to different electricity market participants during gas price spikes and accurately reflect the value of renewables in electricity prices. For example, a well-designed sequential electricity market-clearing scheme may assign greater responsibilities (during gas price spikes) to market participants who have benefited the most from the strong electricity-gas coupling. (3) Specific financial instruments may help to transfer the impacts of gas price spikes to external parties. Although the observations and discussion in the work focus on electricity and gas systems, we believe that risk-hedging contracts offered by financial institutions, such as options to hedge against gas price spikes, have the potential to shift part of the risk from the electricity sector to financial institutions and generate income for these institutions through premiums. The authors would advocate for proactive implementations of electricity-gas coordination and new operation design for New England, rather than relying on the gradual growth of renewable generation to naturally alleviate the situation.

### Limitations of the study

*Limitation 1.* The number of data points on renewable generation and price coupling is from the last five years, limiting the capability to predict the coupling over longer time periods, such as to 2050, when a sudden change in new technologies is possible. Furthermore, the lower bound of renewable generation by 2030 in Figure 2 may deviate from the true value. However, it does not affect the prediction of the limited decreased price coupling.

*Limitation 2.* The percentage in the impact analysis does not reflect the absolute value of the impact of a factor on electricity-gas prices and is not the goal of this article. The magnitude of the percentage is only used to reflect comparisons between different factors.

*Limitation 3.* The simulation studies for electricity-gas cooperation do not consider factors, such as gas infrastructure limitation in the region and accessibility of low-price gas for conversion, which could potentially reduce the effectiveness of electricity-gas cooperation. Furthermore, the electric power flow and gas flow are modeled with linear models, where the dynamics of power and gas flows are not considered. We use marginal cost in the simulation to reflect the electricity-gas price coupling since there is no universal consensus on the pricing scheme in electricity-gas coordination. A detailed description of the simulation system can be found in the supplementary file (Note S3)

*Limitation 4.* The simulation studies for electricity-gas cooperation are only sufficient to show the fact that cooperation can mitigate the asymmetrical influence efficiently, but it does not reflect the actual value of the mitigation ability of cooperation for New England since the reduced system largely simplifies the real-world operation conditions in New England, and the development of renewable integration and PtG technologies may lag or outpace the created scenarios.

## STAR★Methods

### Key resources table

**Table:** 

REAGENT or RESOURCE	SOURCE	IDENTIFIER
**Deposited data**		

Electricity Price	Hourly Day-Ahead LMPs	ISO-New England (https://www.iso-ne.com/isoexpress/web/reports/pricing/-/tree/lmps-da-hourly)
Gas Price	Natural Gas Electric Power Price	EIA Natural Gas (https://www.eia.gov/dnav/ng/ng_pri_sum_dcu_nus_m.htm)
Gas Consumption	Natural Gas Delivered to Consumers	EIA Natural Gas (https://www.eia.gov/dnav/ng/ng_pri_sum_dcu_nus_m.htm)
Gas Consumed (Electric)	Natural Gas Deliveries to Electric Power Consumers	EIA Natural Gas (https://www.eia.gov/dnav/ng/ng_pri_sum_dcu_nus_m.htm)
Gas Price at Henry Hub	Henry Hub Natural Gas Spot Price	EIA Natural Gas (https://www.eia.gov/dnav/ng/ng_pri_sum_dcu_nus_m.htm)
Renewable Generations	Net Generation from Other Renewables	Electricity Data Brower (https://www.eia.gov/electricity/data/browser/)
Gas Generation	Net Generation by Natural Gas	Electricity Data Brower (https://www.eia.gov/electricity/data/browser/)
Electricity Consumption	Net generation for all sectors	Electricity Data Brower (https://www.eia.gov/electricity/data/browser/)

### Resource availability

#### Lead contact

Further information and requests for resources and materials should be directed to and will be fulfilled by the lead contact, Fangxing (Fran) Li (fli6@utk.edu).

#### Materials availability

This study did not generate new unique materials.

#### Data and code availability

• Data: All data used in this paper can be found in the key resources table.
• No new code is generated. The simulation with CURENT LTB is upon requested from the lead contact.
• Any additional information is available from the lead contact upon request.

### Method details

#### Data description

All data used in this study, including electricity/gas prices and generations, are collected from U.S. Department of Energy’s Energy Information Administration (EIA) (https://www.eia.gov/) and ISO New England (https://www.iso-ne.com). The name and source files for all the raw data are listed in key resources table. The discussion on the missing gas price data can be found in Table S1 and Figure S1.

#### Pearson correlation

The Pearson correlation is applied to calculating the electricity-gas price coupling, as shown in Equation 1. The *N* represents the number of prices datapoints. *p*^*e*^ and *p*^*g*^ represent the electricity and gas price, respectively. *μ*^*e*^ and *μ*^*g*^ represents the mean value of electricity and gas price. *σ*^*e*^ and *σ*^*g*^ represent the standard deviation of electricity and gas price.

(Equation 1) $$\rho^{e,g}=\frac{1}{N-1}\sum_{i=1}^{N}\left(\frac{p^{e}-\mu^{e}}{\sigma^{e}}\right)\left(\frac{p^{g}-\mu^{g}}{\sigma^{g}}\right)$$

#### Electricity-gas cooperation model

The IEEE 39 bus system is a well-known simulation system for the New England electric grid, which is used in our study to represent the New England electric grid. More details can be found in the repository (https://icseg.iti.illinois.edu/ieee-39-bus-system/). A simulation system for the gas network in New England is not directly available, and thus we built a simplified gas network in New England based on EIA Energy Altas (https://atlas.eia.gov/). It is worth noting that the reduced system is an approximation and simulation of the electricity-gas cooperation to analyze the electricity-gas price coupling, instead of representing the real operation of physical networks in New England. Three pipelines are considered that are Algonquin Pipeline, Tennessee Pipeline, and Maritime Pipelines. The lower part of the Tennessee Pipeline is not modeled since these areas are covered by Algonquin Pipeline in the simplified system. The topology and simplified systems are performed on Large-scale Testbed^31^^,^^32^ provided by CURENT research center. The electricity-gas coordination model is based on the literature,^45^^,^^46^ where linearized power flow and gas flow models are applied.

### Quantification and statistical analysis

#### Price coupling quantification and predictions

All price coupling values are calculated by Pearson correlation, and the detailed value can be found in Figure 1 and Table S1. From January 2017 to December 2021, renewable generation has moderately increased in VT, RI, and MA, while renewable generation in CT, ME, and NH has stayed at similar levels (Figure S2). We analyzed the coupling values and renewable penetration levels from 2017 to 2021 for VT, MA, and RI. Given the coupling and renewable penetration level values, k-means was used for clustering. The detailed clusters can be found in Table S5. Clustering results, which indicate the staircase relationship between the coupling and renewable penetration levels, are applied to predict the coupling variation in 2030 under MA’s renewable penetration target. The coupling value and the renewable penetration level are regressed through a sinusoidal regression model, and the prediction is made based on the difference between the extreme points (Note S1 and Table S6).

#### Asymmetrical analysis

The examination of the asymmetrical influence between electricity and gas prices is based on a causation analysis. This causation analysis delves into the conditional probability of gas and electricity prices as follows: *For the impact of gas price on electricity price*: Gas prices within the NE region are categorized into two groups (1) higher than the 90^th^ percentile, and (2) lower than the 90^th^ percentile; Electricity prices in the NE region are categorized into three groups (1) less than the 30^th^ percentile; (2) less than the 60^th^ percentile and larger than the 30^th^ percentile; (3) larger than the 60^th^ percentile; The gas reference price at Hery Hub is categorized into two groups: (1) larger than 50^th^ percentile (2) less 50^th^ percentile. *For the impact of electricity price on gas price*: Electricity prices are categorized into two groups by the 90^th^ percentile, instead of the gas price, the gas price is categorized into the three groups similarly, and the gas reference price remains the same. More details on the causation analysis can be found in Table S7. The impact value of different factors on electricity and gas prices can be found in Table S4 (a) and (b), respectively. The share of gas generation in all regions can be found in Table S5. Datasets from March 2006 to December 2021 were collected (as indicated in the key resources table): gas/electricity prices, gas consumption, electricity loads, gas consumed by the electric sector, the share of renewable generation in overall load satisfaction, the share of gas generation in overall load satisfaction, and gas import prices. The electricity and gas prices in the six New England states are fitted through multilinear regression using the above factors. The variation of the R-squared value when a factor is removed from the regression is used to indicate the impact of a factor on electricity and gas prices (Note S2).

## Supplemental information

Document S1. Figures S1–S3, Tables S1–S7, and Notes S1–S3
