# Supplementary material for: Implications of electricity and gas price coupling in US New England region
Source: iScience. 2023 Dec 13;27(1):108726. doi: 10.1016/j.isci.2023.108726 (PMC10792232; doi:10.1016/j.isci.2023.108726)
Supplement: Document S1. Figures S1–S3, Tables S1–S7, and Notes S1–S3 [file mmc1.pdf]

iScience, Volume *27*

Supplemental information

Implications of electricity and gas price coupling in US New England region

Qiwei Zhang, Fangxing Li, Xin Fang, and Jin Zhao

Table S1. Related to Method details. Pearson correlation value between electricity prices in all New England areas and gas prices in MA, CT, and RI, from 2006 to 2021. The gas prices sold to the electric sector in ME, NH, and VT are only partially available to the public due to privacy concerns by the U.S. Department of Energy's Energy Information Administration (EIA). However, the available price data is sufficient for our analysis by measuring the electricity-gas price coupling at ME, NH, and VT through the gas price at CT, MA, and RI since our following results show that the gas price at CT, MA, and RI is tightly coupled with the electricity price at ME, NH, and VT. The discussion of the price coupling at ME, NH, and VT based on the available gas price at ME, NH, and VT are attached as follows. The gas prices sold to the electric sector at ME and NH are only partially available to the public due to privacy concerns by EIA. Over the year 2006 to 2021, there are 16 and 17 months of gas prices data available at ME and NH, respectively. The gas prices sold to the electric sector at VT are only available from 2006 to 2013. The VT has negligible gas-fired generation (i.e., less than 0.1%), and thus the gas prices sold to the electric sector are not applicable in VT by EIA2. The available gas prices sold to the electric sector are plotted in Fig. S1. The coupling values for ME, NH, and VT are 0.83, 0.57, and 0.65 based on the available data. Based on the coupling value, we have observed that the price coupling value between the electricity price and gas price sold to the electric sector in ME, NH, and VT is much lower than the price coupling value between the electricity price in ME, NH, and VT and gas price sold to the electric sector in MA, CT, and RI, which also align with the observation in the article that interconnected electric grid propagates the electricity-gas price coupling from areas where electricity and gas are intensively intertwined to areas where electricity and gas are less intertwined. All correlation values can be found in Table S1.

	Gas Price Sold to Electric Sector		
Electricity price	MA	CT	RI
MA	0.947	0.937	0.945
CT	0.917	0.920	0.917
RI	0.963	0.950	0.964
NH	0.943	0.919	0.960
ME	0.943	0.917	0.960
VT	0.940	0.917	0.958

Table S2. Related to Results. The total electric load consumptions, renewable generations, and nuclear generations for Vermont from 2006 to 2021. The percentage represents the share of renewable generations and nuclear generation in the total electric load consumption. The Pearson correlation is calculated for periods between 2006-2014 and 2015-2021. The Pearson correlation value for electricity and gas price coupling is further calculated for the time periods 2006-2014 and 2015-2020, which are 0.94 and 0.92. Before 2014, VT satisfies over 60% load with renewables and nuclear, but the VT started to rely on electricity import with the close down of nuclear plants after 2014. We did not observe an evident change in the electricity-gas coupling before and after 2014 with the sudden reliance on electricity imports. The electricity-gas price coupling in VT has been tight before 2014. This phenomenon reflects that renewables and nuclear are usually not marginal units to set electricity prices. Therefore, the electricity-gas price coupling did not experience evident changes in VT.

Pearson Correlation	Year	Gas Generation (10^3*MWh)	Hydroelectric Power (10^3*MWh)	Renewable Generations (10^3*MWh)	Nuclear Generation (10^3*MWh)	Electric Load Consumption (10^3*MWh)	Electricity Import (10^3*MWh)	Percentage (%)
0.94	2006	0	14.8	5.9	53.3	82.5	8.3	89.9
	2007	0	6.4	6.1	49.3	70.4	8.5	87.9
	2008	0	14.5	5.7	51.2	80	8.5	89.4
	2009	0.1	14.3	5.8	56.1	84.9	8.7	89.8
	2010	0.1	12.9	6.6	50	77.9	8.3	89.3
	2011	0	13.6	5.8	51.4	79.5	8.6	89.2
	2012	0	10.7	6	52.3	108.3	39.2	63.8
	2013	0	12.3	9.3	50.6	112.3	40.1	64.3
	2014	0	11.2	9.6	52.9	111.8	38.1	65.9
0.92	2015	0	10.6	9.9	0	57.5	36.8	36.0
	2016	0	9.9	9.8	0	50.4	30.6	39.3
	2017	0	11.8	9.9	0	57	35.3	38.1
	2018	0	11.5	10.5	0	55.2	33.2	39.9
	2019	0	11.9	10.6	0	70.7	48.2	31.8
	2020	0	9.9	11.4	0	69.3	48.0	30.7
N/A (Above data for 2021is not fully available on EIA yet)								

Table S3. Related to Results. The total electric load consumptions, renewable generations, and nuclear generations for Vermont from 2006 to 2021. The percentage represents the share of renewable generation and nuclear generation in the total electric load consumption. The Pearson correlation is calculated for periods between 2006-2014 and 2015-2021. The increase of renewable generation in CT, ME, and NH is low, but MA, VT, and RI have increased renewable generation by 10.5%, 5.9%, and 4.5%, respectively, in the last five years (Supplementary Figure 2). The generation resource mix and coupling values of VT are listed in Supplementary Table 2 with Pearson correlation values, and the coupling values and the renewable generation percentages are shown in Table S3. The renewable generation and coupling value in VT are uncorrelated since the Pearson correlation value is negative 0.07, but the Pearson correlation value for the renewable generation and coupling value in MA is as high as negative 0.71. RI shows a low level of correlation with a Pearson correlation value of negative 0.25. Therefore, MA is the only area that indicates a clear correlation between renewable generation and electricity-gas price coupling.

	Massachusetts		Vermont		Rhode Island	
Date	Renewable%	ΔCoupling	Renewable%	ΔCoupling	Renewable%	ΔCoupling
Jan. 2017	7.219	0.968	42.819	0.939	7.632	N/A
Feb. 2017	8.356	0.968	44.542	0.936	8.142	N/A
March 2017	8.617	0.969	40.374	0.937	7.923	0.978
April 2017	6.873	0.969	27.827	0.937	3.547	0.977
May 2017	5.953	0.969	24.563	0.937	5.714	0.977
June 2017	6.666	0.969	34.078	0.938	5.547	0.977
July 2017	6.549	0.969	39.822	0.938	3.476	0.977
Aug. 2017	7.228	0.969	50.61	0.939	3.335	0.978
Sep. 2017	8.349	0.97	52.951	0.939	3.587	0.978
Oct. 2017	7.105	0.97	60.022	0.939	3.366	0.978
Nov. 2017	6.14	0.97	34.836	0.94	4.963	0.978
Dec. 2017	6.361	0.97	46.77	0.937	6.048	0.979
Jan. 2018	6.843	0.971	46.646	0.938	7.055	0.976
Feb. 2018	6.719	0.971	41.376	0.935	7.318	0.974
March 2018	7.524	0.971	41.697	0.935	7.669	N/A
April 2018	6.375	0.971	30.368	0.934	6.337	N/A
May 2018	6.344	0.971	31.12	0.935	4.923	N/A
June 2018	7.254	0.971	52.508	0.935	4.144	N/A
July 2018	7.613	0.971	58.156	0.935	3.327	N/A
Aug. 2018	6.446	0.971	50.547	0.935	2.944	0.974
Sep. 2018	7.514	0.971	48.384	0.935	3.283	0.974
Oct. 2018	8.964	0.97	46.078	0.935	3.592	0.974
Nov. 2018	8.892	0.97	34.48	0.934	6.021	0.974
Dec. 2018	8.734	0.966	35.696	0.933	6.421	0.973
Jan. 2019	7.558	0.965	38.003	0.933	9.271	0.973
Feb. 2019	8.289	0.962	38.679	0.932	10.755	0.969
March 2019	10.101	0.962	37.8	0.932	7.986	0.969
April 2019	12.17	0.961	34.369	0.933	6.753	N/A
May 2019	13.735	0.962	29.906	0.933	5.619	0.969
June 2019	15.231	0.962	31.649	0.934	6.248	0.969

July 2019	8.782	0.962	53.396	0.934	3.769	0.969
Aug. 2019	11.004	0.962	66.852	0.934	3.448	0.970
Sep. 2019	16.546	0.962	68.037	0.935	5.238	0.970
Oct. 2019	13.687	0.962	45.401	0.935	5.218	0.970
Nov. 2019	13.585	0.962	41.88	0.935	7.063	0.970
Dec. 2019	8.949	0.962	41.257	0.936	9.802	N/A
Jan. 2020	9.77	0.959	35.74	0.935	7.632	N/A
Feb. 2020	12.009	0.957	40.314	0.936	8.142	N/A
March 2020	26.883	0.956	37.585	0.936	7.923	N/A
April 2020	23.913	0.955	32.142	0.936	3.547	N/A
May 2020	33.396	0.955	39.479	0.936	5.714	N/A
June 2020	16.035	0.956	63.41	0.935	5.547	N/A
July 2020	9.258	0.956	61.467	0.935	3.476	N/A
Aug. 2020	11.458	0.956	72.529	0.935	3.335	N/A
Sep. 2020	16.509	0.956	78.072	0.936	3.587	N/A
Oct. 2020	12.241	0.956	58.205	0.936	3.366	N/A
Nov. 2020	16.019	0.956	51.905	0.936	4.963	N/A
Dec. 2020	11.313	0.956	44.895	0.936	6.048	N/A
Jan. 2021	9.761	0.956	43.589	0.936	7.055	N/A
Feb. 2021	10.551	0.955	49.042	0.935	7.318	N/A
March 2021	19.502	0.955	52.663	0.935	7.669	N/A
April 2021	25.546	0.955	46.847	0.936	6.337	N/A
May 2021	25.829	0.955	50.666	0.936	4.923	N/A
June 2021	13.685	0.955	53.387	0.935	4.144	N/A
July 2021	14.354	0.955	49.774	0.935	3.327	N/A
Aug. 2021	12.361	0.947	51.215	0.935	2.944	N/A
Sep. 2021	18.106	0.947	46.287	0.934	3.283	N/A
Oct. 2021	16.968	0.947	52.399	0.934	3.592	N/A
Nov. 2021	19.438	0.947	44.517	0.934	6.021	N/A
Dec. 2021	15.725	0.947	52.071	0.933	6.421	N/A
**Pearson Correlation Value**	-0.71		-0.07		-0.25	

Table S4. Related to Method details. The impact value of different factors on electricity price and gas prices.

(a) The impact value of different factors on electricity price

	Factors (%)				
	Gas Prices	Electric Consumptions	Share of Renewables	Share of Gas-fired Generations	Gas Price at Henry Hub
CT	97.5	0.3	0.8	0.3	1.1
MA	98.2	1.3	0.05	0.4	0.05
RI	85.0	2.5	3.4	6.9	1.2
ME	97.6	0	0.2	1.0	1.1
NH	96.8	0.5	0	1.5	1.2
VT	99.2	0.3	0.3	0.1	0.1

(b) The impact value of different factors on gas price

	Factors (%)				
	Gas Prices	Gas Consumptions	Share of Renewables	Share of Gas-fired Generations	Gas Price at Henry Hub
CT	1.7	11.8	11.7	4.5	70.3
MA	2.2	2.3	10.4	23	65.0
RI	0.2	1.4	0.7	2.3	95.3
ME	11.4	1.2	3.0	1.3	83.2
NH	10.5	0.1	0	3.0	86.4
VT	4.1	0.2	4.2	1.6	89.9

Table S5. Related to Method details. The variation of share of gas-fired unit generation in overall electricity generation from 2006 to 2021 for Massachusetts, Connecticut, and Rhode Island.

	Share of Gas Generation (%)		
Year	Massachusetts	Connecticut	Rhode Island
2006	52.298	31.02	96.704
2007	52.458	30.065	97.064
2008	50.132	26.69	97.357
2009	53.611	31.495	97.776
2010	59.175	34.786	97.933
2011	68	45.369	98.232
2012	68.383	45.644	98.446
2013	63.445	44.569	97.591
2014	58.525	43.973	93.877
2015	63.352	46.479	93.468
2016	64.472	49.405	95.407
2017	68.406	46.304	93.839
2018	66.548	50.694	93.389
2019	71.463	53.257	92.987
2020	74.316	57.424	92.647
2021	75.521	55.458	90.044
**Drop (%)**	-23.33	-24.44	6.66

Table S6. Related to Method details. The staircase shape of all clusters.

	Clustered Time Period	Length% (Variation of Renewable generations)	Width (Decreased coupling value)
Cluster 1	Jan. 2017-March 2019	3.0%	0.3%
Cluster 2	April 2019-May 2020	9.0%	0.7%
Cluster 3	June 2020-Dec. 2021	24.1%	0.7%

Table S7. Related to Results. The table of causation analysis, This analysis presents a straightforward examination of the mutual impact between electricity and gas prices. This is supporting evidence for the observation outlined in finding 3 of the paper. This causation analysis delves into the conditional probability of gas and electricity prices, and we established the following framework. *For the impact of gas price on electricity price*: Gas prices within the NE region are categorized into two groups (1) higher than the 90th percentile, and (2) lower than the 90th percentile; Electricity prices in the NE region are categorized into three groups (1) less than the 30% percentile; (2) less than the 60% percentile and larger than the 30% percentile; (3) larger than the 60% percentile; The gas reference price at Hery Hub is categorized into two groups: (1) larger than 50% percentile (2) less 50% percentile. *For the impact of electricity price on gas price*: Electricity prices are categorized into two groups by the 90% percentile, instead of the gas price, the gas price is categorized into the three groups similarly, and the gas reference price remains the same. As shown in Table S8, it is evident that conditioning on high gas prices coinciding with low gas reference prices, the likelihood of high electricity prices significantly increases (demonstrated by a sharp increase from group 1 to group 3). On the contrary, when high electricity prices align with low gas reference prices, the probability of encountering high gas prices only experiences a small rise (with a slight increment from group 1 to group 3). This discrepancy shows that the causal effect of gas prices on electricity prices is stronger than the reversed causal influence. This result aligns with finding 3 showing an uneven mutual impact between electricity and gas prices. This could potentially be due to the fact that the natural gas market is more integrated nationally or globally and the low gas consumption from electric sections in NE may be a small part of overall gas demand, but the electric section strongly depends on the local gas prices.

State	$P(\pi^E=\text{Group}^i \mid \pi^G=\text{High}, \pi^{ref}=\text{Low}), i \in \{1,2,3\}$			$P(\pi^G=\text{Group}^i \mid \pi^E=\text{High}, \pi^{ref}=\text{Low}), i \in \{1,2,3\}$		
	Group 1	Group 2	Group 3	Group 1	Group 2	Group 3
MA	0	0	34%	0	0	15%
CT	0	0	29%	0	0	16%
RI	0	4%	54%	0	4%	17%
NH	2%	10%	34%	2%	10%	16%
ME	3%	1%	53%	0	8%	16%
VT	3%	2%	58%	0	8%	13%

Figure S1. Related to Method details. Natural gas prices sold to the electric sector in all New England areas.

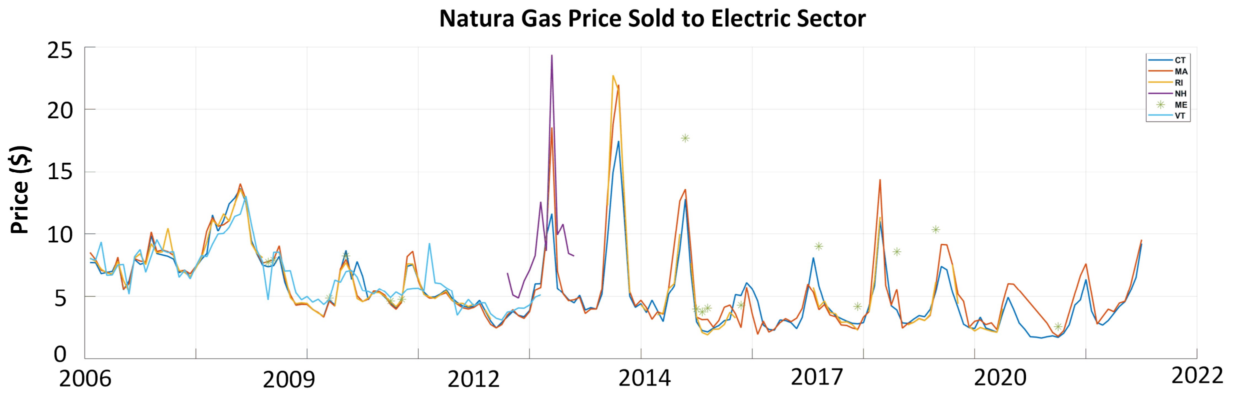

Figure S2. Related to Method details. The renewable generation share in the overall electricity generation across the New England areas. (a) renewable generation shares for Massachusetts. (b) renewable generation shares for Connecticut. (c) renewable generation shares for Maine. (d) renewable generation shares for New Hampshire. (e) renewable generation shares for Vermont. (f) renewable generation shares for Rhode Island. From (a)-(f), the increase of renewable generation in CT, ME, and NH is low, but MA, VT, and RI have increased renewable generation by 10.5%, 5.9%, and 4.5% in the last five years, respectively.

(a)

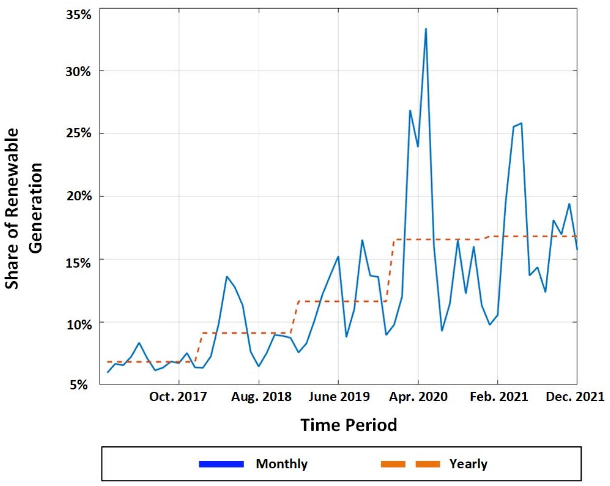

(b)

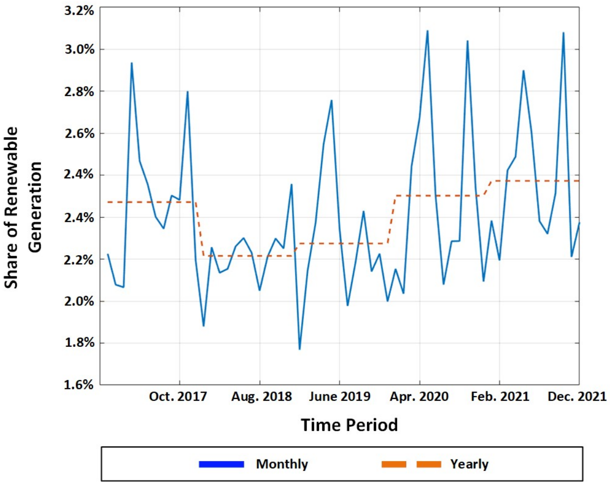

(c)

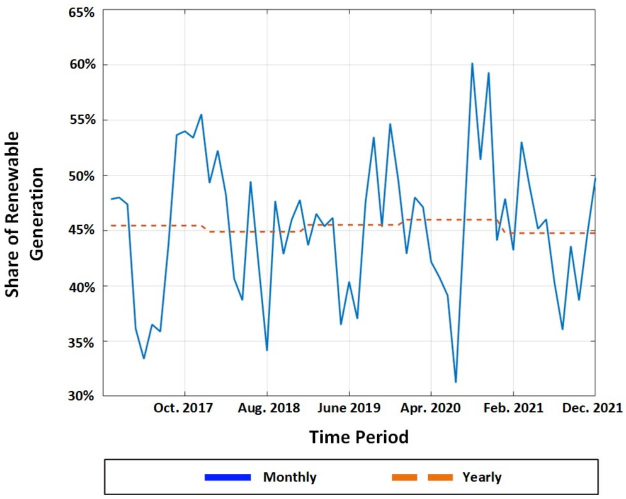

(d)

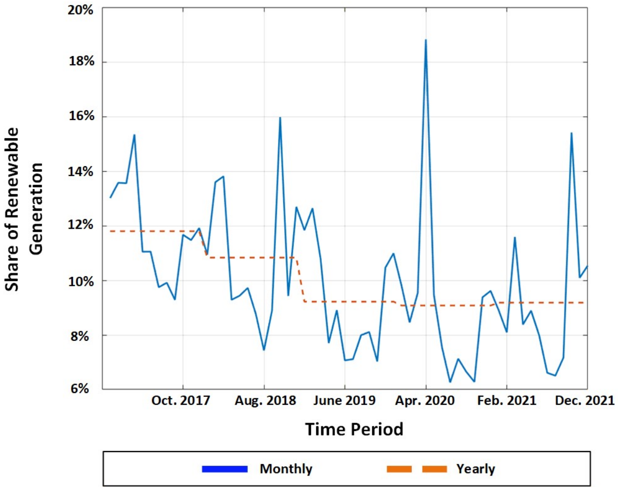

(e)

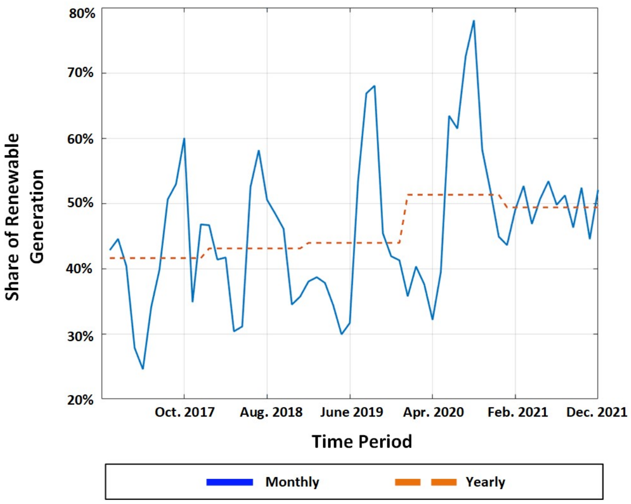

(f)

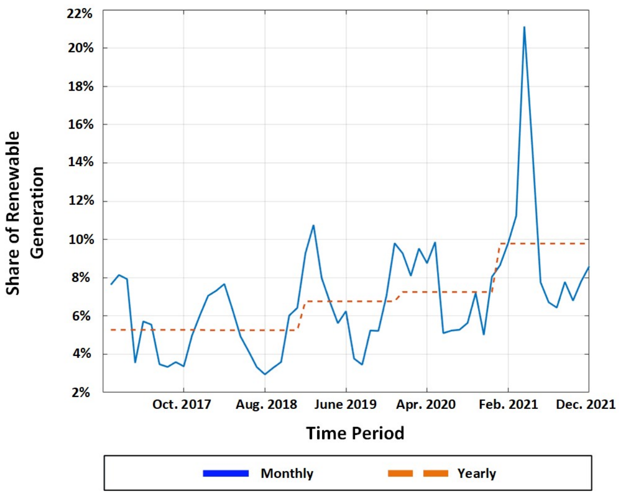

Figure S3. Related to Results The gas consumption from the electric sector and gas prices sold to the electric sector from 2006 to 2021 are normalized and plotted in this figure for CT, MA, and RI.

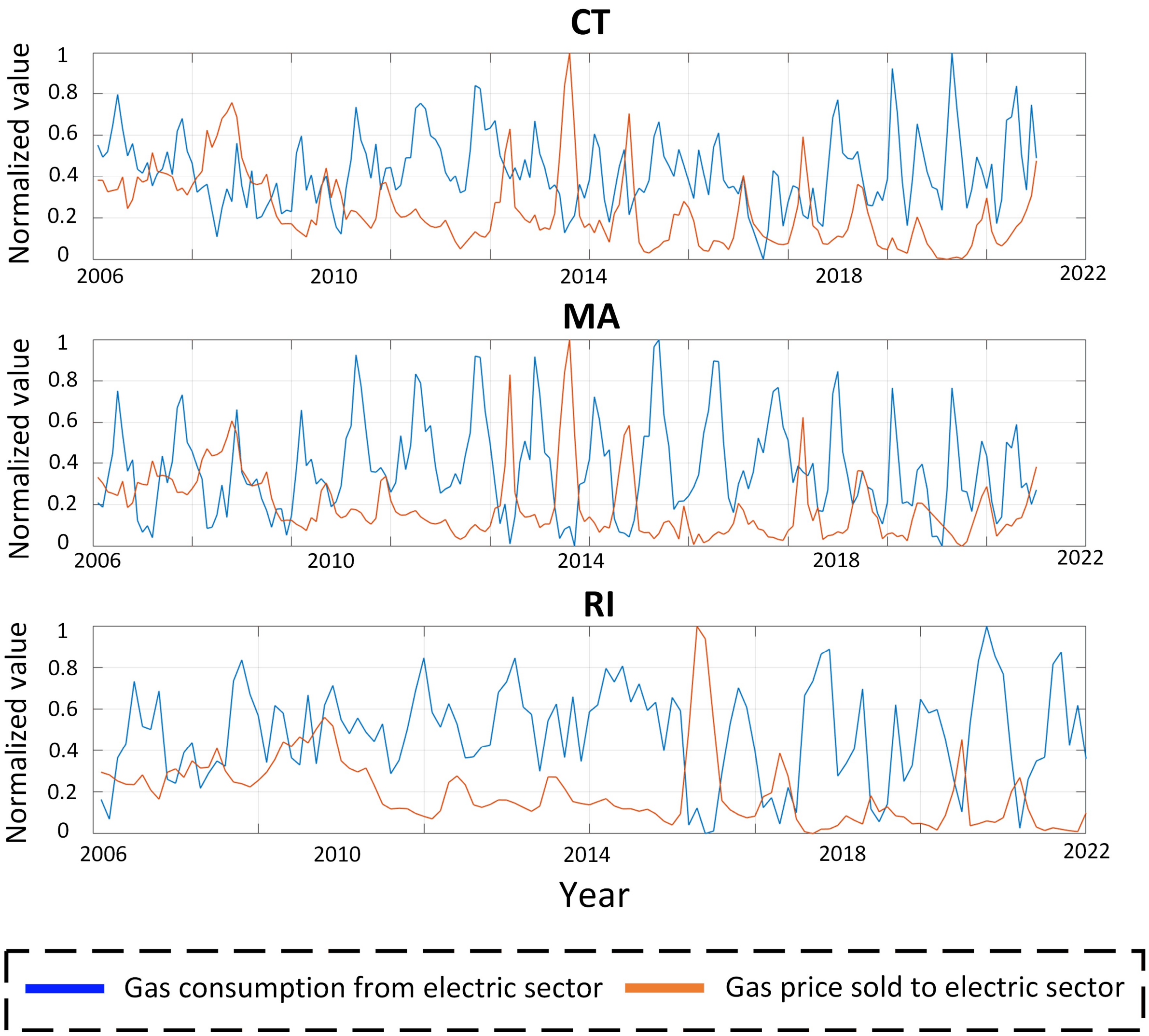

Note S1. Related to Method details. From January 2017 to December 2021, renewable generation has moderately increased in VT, RI, and MA, while renewable generation in CT, ME, and NH has stayed at similar levels (Fig. S2). We analyzed the coupling values and renewable penetration levels from 2017 to 2021 for VT, MA, and RI. Given the coupling and renewable penetration level values, k-means was used for clustering. The detailed clusters can be found in Table S6. The distance metric used in this study is the Euclidean distance, and the elbow method is used to determine the number of clusters. Clustering results, which indicate the staircase relationship between the coupling and renewable penetration levels, are applied to predict the coupling variation in 2030 under MA's renewable penetration target. The coupling value and the renewable penetration level are regressed through a sinusoidal regression model (1). The staircase shape can be calculated by the difference between the extreme points, as in eq. (2)

$$cop^{predict} = \sum_{i=1}^{n} a_i \sin(b_i x + c_i) \quad (1)$$

$$len = x_i^u - x_i^l, \forall i \in \{\text{groups}\}, \forall x \in \{g_i^{res}, cop_i\} \quad (2)$$

Note S2. Related to Method details. The impact value of different factors on electricity and gas prices can be found in Supplementary Table 4 (a) and (b), respectively. It is worth noting that the impact value of gas price on electricity price is relatively lower in RI. We believe the reason is that the high share of gas generation has experienced around a 7% drop in RI. The share of gas generation in CT and MA has increased by over 20%, and the share of gas generation in ME has decreased from 20%, the share of gas generation in VT and NH remains at a similar level. The share of gas generation in all regions can be found in Supplementary Table 5. It reflects that the increase in the share of gas generation from a medium value to a high value or decrease from a medium value to a low value barely affects the existing strong impact of gas prices on electricity prices, but it will impose certain impacts if the share decreases from a high value. It should be noted that electricity-gas price coupling is still strong in RI, the impact value only reflects those other factors has affected the variation of gas prices. Datasets from March 2006 to December 2021 were collected: gas/electricity prices, gas consumption, electricity loads, gas consumed by the electric sector, the share of renewable generation in overall load satisfaction, the share of gas generation in overall load satisfaction, and gas import prices. The source data of each factor in the impact analysis is from EIA and ISO-NE. These data are too massive to be included in the supplementary file, and thus we provide the name of the raw data and source file for each factor in Supplementary Table 5. The electricity and gas prices in the six New England states are fitted through multilinear regression using all of the above factors. The variation of the R-squared value when a factor is removed from the regression is used to indicate the impact of a factor on electricity and gas prices. The impact in terms of percentage is calculated by equation (3), where R_{all}^{sq} and R_i^{sq} represent the R-squared value when all factors are considered in the regression and the R-squared value when a factor is removed from the regression.

$$I^f = \frac{R_{all}^{sq} - R_i^{sq}}{\sum_{i=1}^{N} R_{all}^{sq} - R_i^{sq}} \quad (3)$$

Note S3. Related to Method details. The IEEE 39 bus system is a well-known simulation system for the New England electric grid, which is used in our study to represent the New England electric grid. More details can be found in literature[5,6]. A simulation system for the gas network in New England is not directly available, and thus we built a simplified gas network in New England based on EIA Energy Altas[7]. It is worth noting that the reduced system is an approximation and simulation of the electricity-gas cooperation to analyze the electricity-gas price coupling, instead of representing the real operation of physical networks in New England. Three pipelines are considered that are Algonquin Pipeline, Tennessee Pipeline, and Maritime Pipelines. The lower part of the Tennessee Pipeline is not modeled since these areas are covered by Algonquin Pipeline in the simplified system. The topology and simplified systems are performed on Large-scale Testbed[Error! Reference source not found.] provided by CURENT research center. The electricity-gas coordination model is based on the literature[Error! Reference source not found.], where linearized power flow and gas flow models are applied.

References

1. ISO-NE, Day-Ahead LMPs, https://www.iso-ne.com/isoexpress/web/reports/pricing/-/tree/lmps-da-hourly
2. EIA, Natural Gas Prices, https://www.eia.gov/dnav/ng/ng_pri_sum_dcu_nus_m.htm
3. EIA, Electricity Data Browser, https://www.eia.gov/electricity/data/browser/
4. Bai, L., Li, F., Cui, H., Jiang, T., Sun, H., & Zhu, J. (2016). Interval optimization based operating strategy for gas-electricity integrated energy systems considering demand response and wind uncertainty. Applied energy, 167, 270-279. https://doi.org/10.1016/j.apenergy.2015.10.119.
5. Illinois Center for a Smarter Electric Grid, IEEE 39-Bus System, https://icseg.iti.illinois.edu/ieee-39-bus-system/
6. Electric Grid Test Case Repository, New England IEEE 39-Bus System,

https://electricgrids.engr.tamu.edu/electric-grid-test-cases/new-england-ieee-39-bus-system/
7. EIA, U.S. Energy Atlas. https://atlas.eia.gov/
8. Parsly, Nicholas, et al. (2022). DiME and AGVIS A Distributed Messaging Environment and Geographical Visualizer for Large-scale Power System Simulation. preprint at arXiv. https://doi.org/10.48550/arXiv.2211.11990.
